# Tunnel engineering for modulating the substrate preference in cytochrome P450_Bsβ_HI

**DOI:** 10.1186/s40643-021-00379-1

**Published:** 2021-04-03

**Authors:** Shuaiqi Meng, Ruipeng An, Zhongyu Li, Ulrich Schwaneberg, Yu Ji, Mehdi D. Davari, Fang Wang, Meng Wang, Meng Qin, Kaili Nie, Luo Liu

**Affiliations:** 1 Beijing Bioprocess Key Laboratory, Beijing University of Chemical Technology, Beijing, 100029 People’s Republic of China; 2 Institute of Biotechnology, RWTH Aachen University, Worringerweg 3, 52074 Aachen, Germany; 3 DWI-Leibniz Institute for Interactive Materials, Forckenbeckstraße 50, 52074 Aachen, Germany

**Keywords:** Tunnel engineering, Substrate preference, Cytochrome P450_Bsβ_HI, α-Alkene biosynthesis, Rational design

## Abstract

An active site is normally located inside enzymes, hence substrates should go through a tunnel to access the active site. Tunnel engineering is a powerful strategy for refining the catalytic properties of enzymes. Here, P450_Bsβ_HI (Q85H/V170I) derived from hydroxylase P450_Bsβ_ from *Bacillus subtilis* was chosen as the study model, which is reported as a potential decarboxylase. However, this enzyme showed low decarboxylase activity towards long-chain fatty acids. Here, a tunnel engineering campaign was performed for modulating the substrate preference and improving the decarboxylation activity of P450_Bsβ_HI. The finally obtained BsβHI-F79A variant had a 15.2-fold improved conversion for palmitic acid; BsβHI-F173V variant had a 3.9-fold improved conversion for pentadecanoic acid. The study demonstrates how the substrate preference can be modulated by tunnel engineering strategy.

**Supplementary Information:**

The online version contains supplementary material available at 10.1186/s40643-021-00379-1.

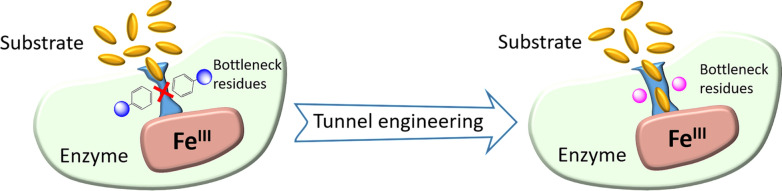

## Introduction

Enzymes are able to catalyze many specific reactions and are widely used in practical application. Previously, the enzyme functions were limited around their natural use; nowadays, the enzymes could be developed by engineering their activity and selectivity for meeting human demands (Bornscheuer et al. 2012; Damborsky and Brezovsky 2014). Two common engineering strategies are the directed evolution based on Darwinian theory and rational design based on the structure–function relationship (Bornscheuer and Pohl 2001). Rational design often focused on the substrate binding pocket which directly influences the enzymatic process (Bornscheuer and Pohl 2001). However, the experiences of directed evolution told us the residues outside the active site also influence the enzyme properties (Kress et al. 2018). Researching on those “non-hotspot” rather than typical active site may be beneficial for expanding the understanding of proteins.

The urgent problem now confronting us is how to obtain the important “non-hotspot” domains beyond the enzyme active site. Tunnel engineering may be one of the answers. Enzymes spanning all of the six classes are found to exist of the tunnels (Kingsley and Lill 2015). It was reported that more than 64% enzymes annotated in Catalytic Site Atlas library have the buried active site with the tunnels connecting the enzyme binding pocket and the environment (Pravda et al. 2014). The tunnels could support the transport of solvent, product and solvent between the enzyme active site and bulk solvent, which play important role in enzymatic reaction (Kokkonen et al. 2019; Zhou and McCammon 2010). The behavior of substrate on the tunnels to the active site could affect the activity, stability and substrate selectivity (Kingsley and Lill 2015; Lu et al. 2019; Yu et al. 2013). A typical example might be that the R47 and Y51 residues, two polar amino acids located at the end of access tunnel of P450 BM3, which could regulate the entry of substrate, water and co-solvents (Whitehouse et al. 2012). Cheng et al. found that a single position located on the access tunnel of nitrile hydratase could invert the regio-selectivity towards aliphatic *α*,*ω*-dinitriles (Cheng et al. 2016). Bauer et al. narrowed the substrate tunnel by replacing a valine by a larger isoleucine causing a significantly improved catalytic efficiency of decarboxylase P450_OleT_ towards short-chain fatty acid (Bauer, et al. 2020). Tunnel engineering is becoming a promising strategy to optimize the enzyme property. Several scientists have developed many algorithms for determination of enzymatic tunnels, such as CAVER (Kozlikova et al. 2014), MOLE (Sehnal et al. 2013), AQUA-DUCT (Magdziarz et al. 2017) and CCCPP (Benkaidali et al. 2014). However, compared with the prediction of tunnels, only a few experimental tunnel engineering examples were reported (Kress et al. 2018). Here, we aim to develop the application of tunnel engineering for modulating the substrate preference.

α-Alkenes are multifunctional compounds that perform an important industrial value and an extraordinary economic importance due to their flexible and active chemical performance. Particularly, long-chain α-alkenes can be used in the synthesis of high-value biofuel, lubrication and surfactant (Lee et al. 2008). However, α-alkenes mostly come from non-renewable petroleum cracking (Dutta et al. 2014). Energy-intensive process and harsh reaction conditions prompted researchers to focus on enzymatic synthetic strategy of α-alkenes (Schirmer et al. 2010). To date, three biotransformation strategies that convert fatty acids or its derivative to alkene were reported (Yi et al. 2014). A three-gene cluster from *Micrococcus luteus* represented a production of long-chain alkenes from a head-to-head condensation of fatty acids (Beller et al. 2010); type I polyketide synthases from *Synechococcus* sp*.* were involved in a production of medium-chain α-alkenes via an elongation decarboxylation mechanism (Mendez-Perez et al. 2011); and some members from cytochrome P450 family are able to decarboxylate fatty acids and produce α-alkenes directly (Grant et al. 2015; Hsieh and Makris 2016). Among those pathways, the decarboxylic reaction catalyzed by P450 is the simplest strategy. Free fatty acids could be used as substrate directly, which represent potential of producing α-alkenes on a large-scale based in engineered cell factory.

Representative P450 decarboxylases belong to CYP152 subfamily, such as P450_Bsβ_ (CYP152A1) from *Bacillus subtilis* (Matsunaga et al. 1999), P450_Spα_ (CYP152B1) from *Sphingomonas paucimobilis* (Matsunaga et al. 2000), and P450_oleT_ (CYP152L1) from *Jeotgalicoccus sp.* (Yi et al. 2014). CYP152 family could utilize H_2_O_2_ as an oxidant, which is miscible with water and easy to directly add to the reaction than O_2_ employed by most of P450 monooxygenases (Jiang et al. 2019). P450_oleT_ is a particularly remarkable member among CYP152 family since it presented prominent decarboxylation property, which is able to convert medium- or long-chain fatty acids to corresponding carboxylic acids (Rude et al. 2011). However, P450_oleT_ shows severely inactivated under millimole concentrations of H_2_O_2_ (Dennig et al. 2015). In contrast, P450_Bsβ_ has relatively high H_2_O_2_ tolerance (Wang, et al. 2020). As the first member of CYP152 subfamily, P450_Bsβ_ was the research hotspot since it was discovered by Isamu et al*.* in 1999, although wild-type P450_Bsβ_ showed higher hydroxylation activity rather than decarboxylation activity (Matsunaga, et al. 1999). Interestingly, some P450_Bsβ_ variants also exhibited satisfactory decarboxylation ability. Using directed evolution method, Wang et al. evolved a P450_BSβ_ variant (V74I/Q85H/F166M/G290V) which showed high reactivity towards saturated and unsaturated fatty acids (Wang et al. 2020). Xu et al*.* reported a P450_Bsβ_HI variant (Q85H/V170I) which displayed enhanced decarboxylation activity towards medium- or long-chain fatty acids (Xu et al. 2017). However, the yield of α-alkenes drops sharply as the length of the carbon chain of the substrate increases, which is the main limitation of the use of P450_Bsβ_ for long-chain α-alkenes synthesis.

In the present work, the cytochrome P450_Bsβ_HI was used as research model. We systematically analyzed the access tunnels in this enzyme. In order to improve the decarboxylation activity of P450_Bsβ_HI to long-chain fatty acids, two residues related to the access tunnels diameter were identified and mutated. In addition, the substrate selection mechanism controlled by tunnels of P450_Bsβ_HI was briefly discussed.

## Experimental

### Strain, plasmid and chemicals

*Escherichia. coli* strain BL21(DE3) cells (used for gene expression) and TOP10 cells (used for molecular cloning) were purchased from TransGen Ltd. (China) The gene P450_Bsβ_HI (derived from P450_Bsβ_ (NCBI Reference Sequence: WP_119898938.1) with a Q85H/V170I mutagenesis) was synthesized by Inovogen Ltd. (China). Fast Mutagenesis kit was from Vazyme Biotech Co., Ltd (China). Plasmid extraction kits and gel extraction kits were obtained from Omega Bio-tek (USA). Capric acid, lauric acid, myristic acid, stearic acid, undecene and tridecene were purchased from BioRo Yee Ltd. (China). All chemicals used were of analytical grade.

### Molecular modeling and simulation

The structure model of P450_Bsβ_HI was constructed by PyMoL Molecular Graphics System (version 2.3.3) based using the crystal structure of P450_Bsβ_ from *Bacillus subtilis* (PDB code: 1IZO with the resolution in 2.10 Å) as template (Lee et al. 2003). Molecular dynamics simulation was carried out in YASARA (version 17.8.15) using the built-in MD macro “md_run.mrc” with the AMBER03 force field (Hess et al. 2008). Structure was solvated into a 12 Å cube simulation cell of water molecules. The box was filled with 3857 water molecules. The simulations of the protein–water system was performed at 303 K, pH value of 8.0. Na^+^ and Cl^−^ were used to neutralize the systems. In all simulations, constant pressure periodic boundary conditions were used for 5 ns MD production. The simulation snapshots were captured every 100 ps from 2.5 ns to 5 ns (after RMSD stabilizes).

### Tunnel analysis

Tunnel analyzed by MOLEonline (Pravda et al. 2018) with following parameters: interior threshold 1.1 Å, bottleneck tolerance 3 Å, bottleneck radius 1.2 Å. Probe radius 5 Å, surface cover radius 10 Å. The starting point was the heme co-factor. Water molecules were not considered in tunnel analysis.

Tunnels analyzed by Caver Analyst 2.0 BETA (Jurcik et al. 2018) with following procedures: after the MD simulation, 25 snapshots were introduced to Caver Analyst 2.0 BETA. Tunnel calculations were performed with the analytical parameters as follows: probe radius of 1.4 Å, shell depth of 4 Å, shell radius of 3 Å, clustering threshold of 3.5, and the starting point of surrounding the residues of R242, P243, and heme. After the tunnel calculations, the influence of the tunnel by related residues was extracted from the function of tunnel statistics and residue graph. Finally, amino acid residues with significant influence on the tunnel bottleneck were selected for further mutagenesis experiments.

### Evolutionary conservation analysis of P450_Bsβ_HI

The evolutionary conservation of P450_Bsβ_HI residues were analyzed by the ConSurf server (https://consurf.tau.ac.il/) (Ashkenazy et al. 2010, 2016; Celniker et al. 2013). The homolog search algorithm was HMMER, and the proteins database was UNIREF-90. There were 150 sequences used for a Multiple Sequence Alignment with the alignment method of MAFFT-L-INS-i.

### Relative folding free energies (ΔΔG_fold_) analysis

The ΔΔG_fold_ values were calculated using FoldX employing the YASARA plugin (version 19.12.4). The structure model of P450_Bsβ_HI and its variants were constructed by PyMoL Molecular Graphics System (version 2.3.3) based using the crystal structure of P450_Bsβ_ from *Bacillus subtilis* (PDB code: 1IZO with the resolution in 2.10 Å) as template. The initial structure of P450_Bsβ_HI was constructed by the Automated Modeling Tool of Swiss Model Web Service (http://swissmodel.expasy.org/) using the crystal structure of P450Bsβ from *Bacillus subtilis* (PDB code: 1IZO with the resolution in 2.10 Å) as template. FoldX parameters were temperature 298 K, pH 8, and 0.05 M ionic strength.

### Molecular docking

Ligand lauric acid was created and minimized in YASARA (version 19.12.14). A grid box of 8 Å around the active site was generated by centering the heme iron of P450_Bsβ_HI or its variants. Molecular docking simulations were performed in YASARA using the built-in macro “dock_run.mrc”. Docking method was VINA with a fixed protein backbone. 25 docking runs were performed for each variant, and the docking poses were clustered by applying a RMSD cutoff of 5 Å and using the default settings provided within the YASARA dock_run macro file.

### Site-specific mutagenesis

The P450_Bsβ_HI gene was inserted into plasmid pET22b( +) between the NdeI and HindIII restriction nuclease sites, with the His-tag encoding sequence at N-terminal. All of the variants were done by Fast Mutagenesis kit.

The variants with single mutation used P450_Bsβ_HI as template, in which F79 and F173 sites were individually replaced with relatively small amino acids, including glycine, valine, alanine, serine, isoleucine, threonine, cysteine, leucine, and proline. Variants with double mutations used BsβHI-F173V variant as template.

### Heterologous expression and purification

P450_Bsβ_HI and its variants were transferred to *E. coli* BL21(DE3) for expression. Recombinant *E. coli* BL21 (DE3) cells were grown in LB medium (5 g/L yeast extract, 10 g/L tryptone, and 5 g/L NaCl) supplemented with ampicillin (100 µg/ml) at 37 ℃ until the OD600 reached about 0.6. All of genes were induced by the addition of 0.1 mM of isopropyl-β-d-thiogalactopyranoside (IPTG) at 18 ℃ for 12 h. Then the cells were harvested and ultrasonically broken. Cell-free extractions were used for purification.

Enzymes were purified by His-tag affinity chromatography. Cell-free extractions was loaded onto a Ni–NTA column and equilibrated by lysis buffer (50 mM of Tris–HCl, 10 mM of imidazole and 300 mM of NaCl, pH 7.8). Then the protein was sequential eluted by wash buffer (50 mM of Tris–HCl, 30 mM of imidazole and 300 mM of NaCl, pH 7.8) and elution buffer (50 mM of Tris–HCl, 300 mM of imidazole and 300 mM of NaCl, pH 7.8). Eluates were concentrated with an ultrafiltration (Millipore, Germany). The concentrations of purified proteins were determined by BCA kit (Solarbio, China).

### Enzymatic assay

The bio-catalytic system contained 10 µM P450_Bsβ_HI enzyme (or its variants), 500 µM fatty acid substrate (from a 100 mM stock solution in ethanol) and 1 mM H_2_O_2_ in a final volume of 1 mL of 100 mM potassium phosphate buffer (pH = 8.0). The reaction was carried out at 30 ℃ for 2 h. After reaction, 200 µM of the tridecene was added as internal standard compound (particularly, undecene was used as internal standard when using myristic acid as the substrate), then additional 50 µL of 6 M HCl was used for reaction quenching. The mixture was extracted by 800 μL hexane. Following extraction, the alkene products were analyzed by gas chromatography–mass spectrometry (GC–MS-QP2020, Shimadzu, Japan) equipped with a Sh-Rxi-5Sil-Ms column (Shimadzu, Japan) using helium as carrier gas. In particular, for detection of hydroxyl fatty acid products, the extracted samples were additionally derivatized with 100 μL *N*-methyl-*N*-(trimethylsilyl)trifluoroacetamide (MSTFA) at 50 ℃ for 2 h before GC–MS analysis. The oven temperature was controlled initially at 50 ℃ for 2 min, then increased at the rate of 10 ℃ min^−1^ to 280 ℃, and held for 10 min. The injecting temperature was 280 ℃. The concentration of alkene and hydroxyl fatty acid products was calculated by calibration curves with internal standards (Additional file 1: Figure S5).

## Results and discussion

The result and discussion part is divided into three parts. In the first part, the P450_Bsβ_HI access tunnels were analyzed and two amino acids were identified as beneficial key residues. In the second part, the variants based on the two key residues were characterized with lauric acid substrate then 5 best variants were selected. In the third part, the substrate preference of selected P450_Bsβ_HI variants was investigated.

### P450_Bsβ_HI access tunnel analysis and hot spots identification

**Fig. 1:**
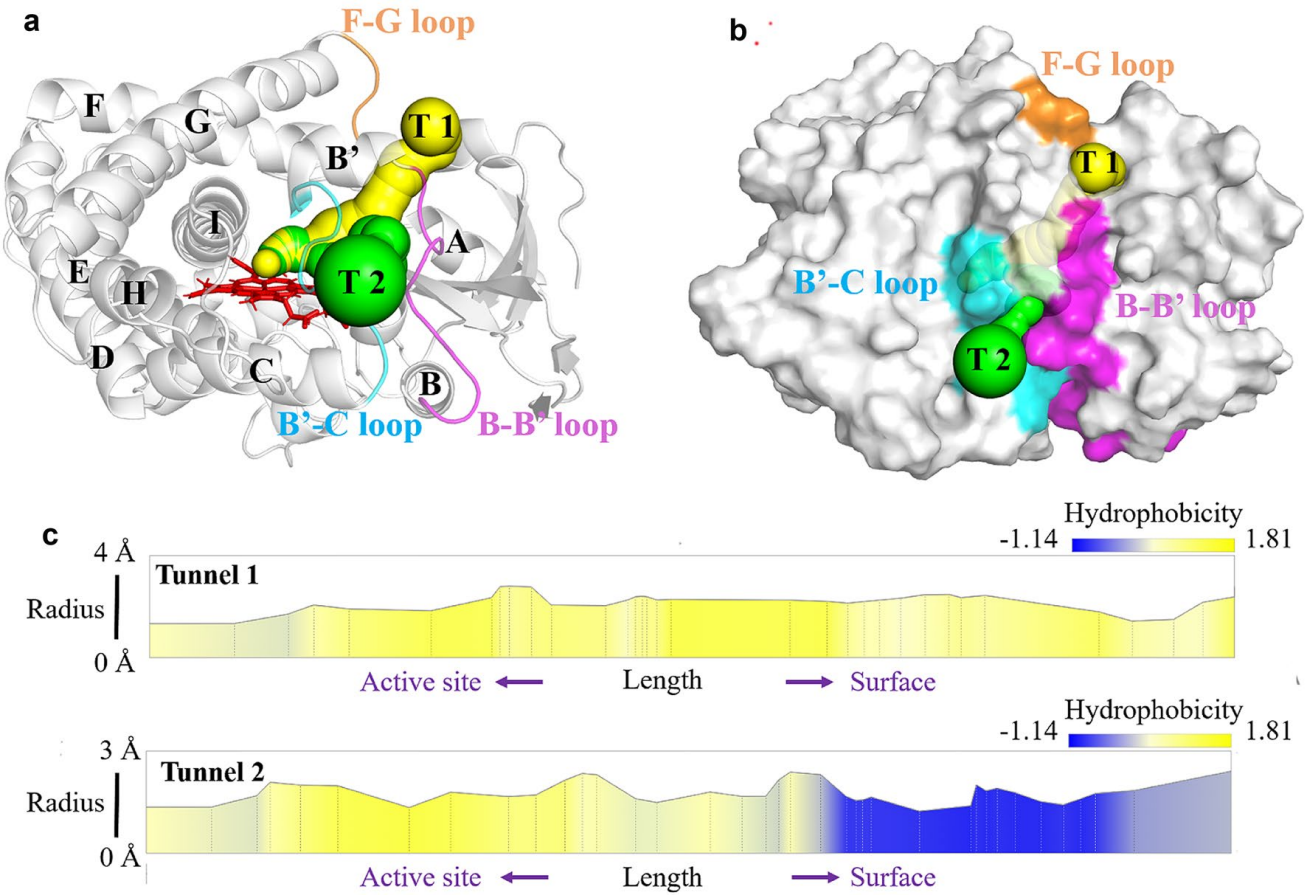
The overall structure and substrate access tunnels information of P450_Bsβ_HI. The structure model of P450_Bsβ_HI was constructed by PyMoL based on the crystal structure of P450Bsβ (PDB: 1IZO). The access tunnel identification and hydrophobicity analysis were carried out with MOLEonline (Pravda, L, 2018). **a** and **b** The structure of the P450_Bsβ_HI enzyme. Access tunnel 1 (T1) and 2 (T2) were colored in yellow and green, respectively. The B-B’ loop, B’-C loop and F-G loop were colored in orange, purple and cyan, respectively, **c** The hydrophobicity property of identified tunnels. Hydrophobicity index according to Hilda Cid et al from the charged residues (Glu -1.14) to nonpolar residues (Ile 1.81) (Cid et al. 1992)

Access tunnels are responsible for ligand transportation between active site and solvent environment in enzymes with buried active site (Kokkonen et al. 2019). Using MOLEonline server (Pravda et al. 2018), two access tunnels were found in P450_Bsβ_HI (Fig. 1a, b and Table 1). The tunnels showed typical cytochrome features. The F and G helix define the most common tunnels among cytochrome P450s (Cojocaru et al. 2007). The peripheral flexible F-G loop can control the tunnel topology then influence substrate\recognition. Representative tunnel in P450_Bsβ_HI is Tunnel 1, which goes through the A helix, B’ helix, B-B’ loop B’-C loop, and F-G loop. Tunnel 1 consists of a large number of nonpolar residues, which facilitates the access of hydrophobic substrates (Additional file 1: Table S1). Tunnel 2 threads through B-B’ loop and B’-C loop, which is also common among P450s. Compared with Tunnel 1, Tunnel 2 shows higher polarity and contains a hydrophilic area near the surface of P450_Bsβ_HI (Fig. 1c and Table 1). In addition to participating in substrate transport, this tunnel may be also involved the controlling water diffusion.

**Table 1:** The properties of identified access tunnels in P450_Bsβ_HI

Tunnel	Length (Å)	Bottleneck radius (Å)	Polarity*
Tunnel 1	29	1.4	4.06
Tunnel 2	37	1.2	8.48

The access tunnel analysis was carried out with MOLEonline

*Polarity is calculated as an average of amino acid polarities assigned according to the method of Zimmerman et al. (1968). Polarity ranges from completely nonpolar amino acids (ALA, GLY = 0.00) through polar residues (e.g., SER = 1.67) towards charged residues (GLU = 49.90, ARG = 52.00)

**Fig. 2:**
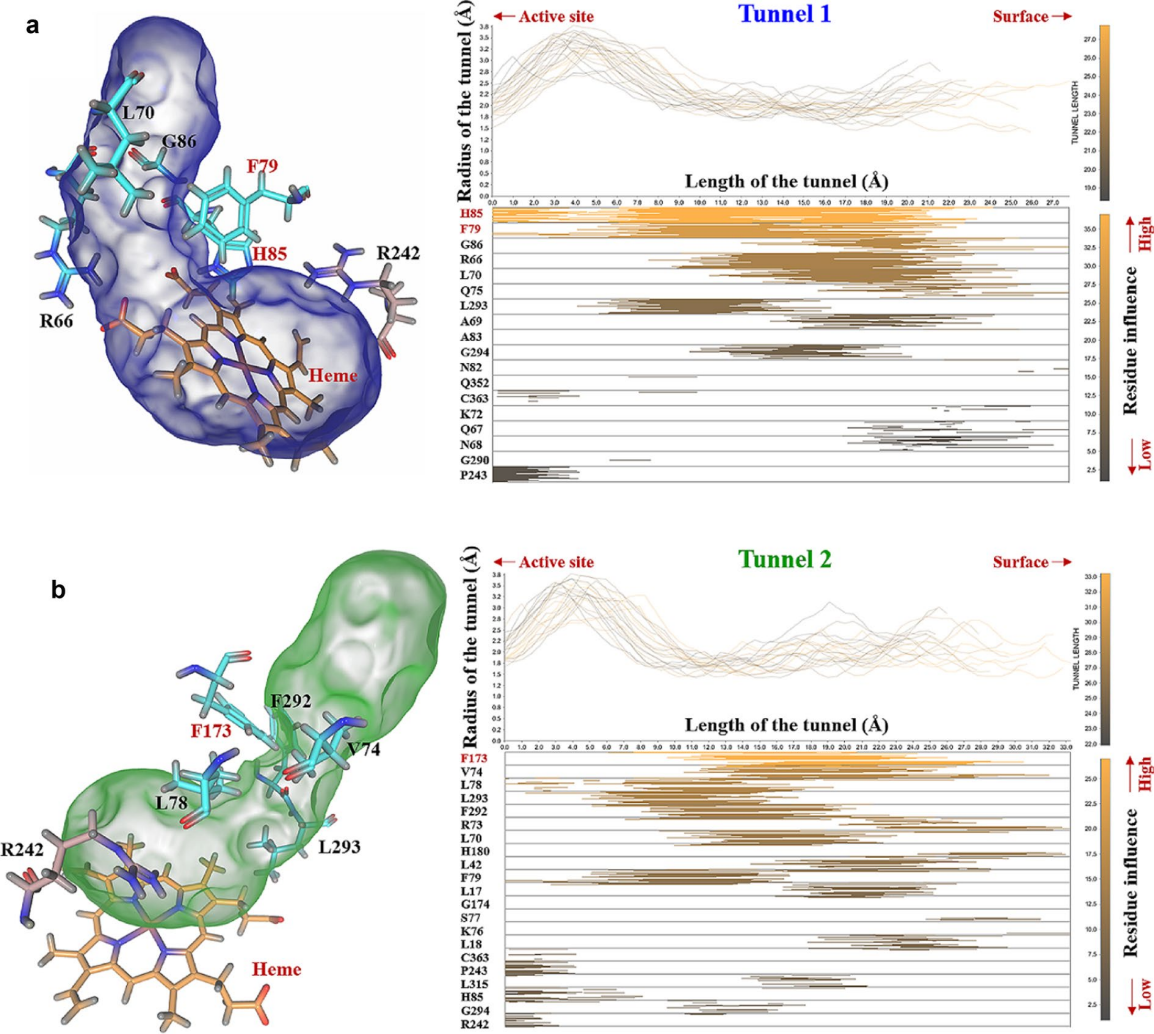
Evolution of residues lining the access tunnels of P450_Bsβ_HI. The access tunnel analysis was carried out with Caver Analyst 2.0 BETA. The left part is bottleneck residues lining of tunnel 1 and 2. The upper right part shows graph representation plots the tunnel width along its centerline. Each line represents the tunnel in one timestep. The lower right part describes the list of all residues contributing to the tunnel. The horizontal position of the color in the line shows which part of the tunnel this residue influences. All tunnel structure information was from the 5-ns snapshot of MD simulation

In order to find potentially important residues, we set four criterions: (1) located in the tunnel bottleneck area; (2) being highly influential along the entire tunnel; (3) located in the loop area; (4) being not completely conserved in its homologous cytochromes. Caver Analyst 2.0 software was chosen for analyzing the residues influence, which provides an opportunity to explore the portion of the tunnel influenced by a particular amino acid (Jurcik et al. 2018). We analyzed the tunnel properties and bottleneck residues influence over 5 ns (Fig. 2). The results indicate that residues of F173 and V74 have high impact on Tunnel 1, and H85 and F79 are the main bottleneck residues in access Tunnel 2. Among high-impact residues, H85 is the core residues for P450_Bsβ_HI decarboxylation activity (Xu et al. 2017), V74 is located in the middle of B’ helix, while the two phenylalanine, F79 and F173, are non-conservative (Additional file 1: Figure S1) and located in the B’-C loop and F-G loop, respectively. Given that, F79 and F173 residues were chosen for further analysis to optimize the enzymatic property while avoid ruining tunnel architecture.

Theoretically, the transport ability of substrates between enzyme active site and solvent environment could be adjusted by gates located at the access tunnels (Gora et al. 2013). Aromatic amino acids in access tunnels bottleneck often participated in the control of the putative gates (Pavlova et al. 2009). As shown in Fig. 2, the huge aromatic side chains of F79 and F173 were involved in the tunnel bottleneck regions. Here, the two phenylalanine residues F79 and F173 are presumed to be the “gatekeeper”, i.e., control the access of substrate from solvent environment to P450_Bsß_HI active site. In addition, the F79 and F173 are also deemed to stabilize the fatty acid substrates via hydrophobic interactions (Lee et al. 2003). Based on the critical location of the two residues, it is assumed the large phenyl side chain of phenylalanine maintains substrate stability, but hinders the entry of long-chain fatty acid substrates. A more flexible substrate entrance may benefit to increase enzyme activity towards long-chain fatty acids substrate. To test the hypothesis, the F79 and F173 residues were replaced by relatively small amino acid including cysteine, isoleucine, leucine, alanine, glycine valine, serine, threonine and proline, in mutagenesis experiments, respectively. The variants stability was evaluated by analysis of the relative free energy of folding (ΔΔG_fold_) (Cui et al. 2020) and no unstable substitution was found (Additional file 1: Figure S2). Variants were heterologously expressed in *E. coil* BL21(DE3).

### Decarbonylation activity of P450_Bsβ_HI variants

**Fig. 3:**
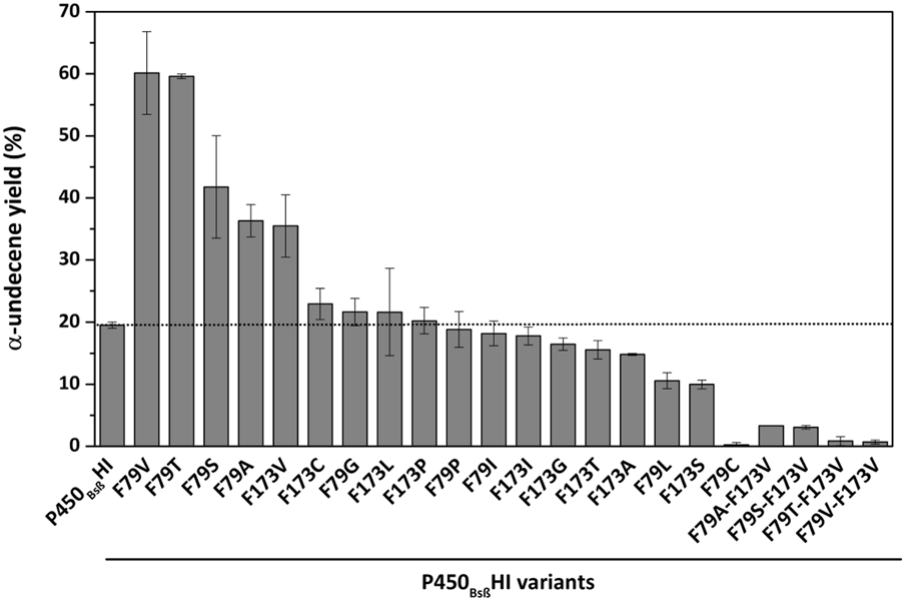
Evolution of the activity of P450_Bsβ_HI variants towards the decarboxylation of lauric acid. The α-undecene products were qualitatively determined by contrasting with standard sample, and quantified by external standard method. Every bio-catalytic system contained 10 µM P450_Bsβ_HI enzyme (or its variants), 500 µM lauric acid substrate (from a 100 mM stock solution in ethanol) and 1 mM H_2_O_2_ in a final volume of 1 mL of 100 mM potassium phosphate buffer (pH = 8.0). Results shown are mean ± SD of duplicated experiments

As a hydrogen peroxide-dependent enzyme, P450_Bsβ_HI relies on H_2_O_2_ as an electrons provider and oxygen supplier (Xu et al. 2017). To select promising variants of P450_Bsβ_HI, we examined the enzyme activity with the substrate of lauric acid using H_2_O_2_ as the sole co-factor. Decarboxylation efficiency was characterized by α-undecene yield. As shown in Fig. 3, 9 of 18 single-site variants showed improved yield of α-undecene. Particularly, BsβHI-F79A, BsbHI-F79S, BsβHI-F79T, BsβHI-F79V, and BsβHI-F173V exhibited more than 1.5-fold increase of α-undecene yield compared to P450_Bsβ_HI. The optimal variant BsβHI-F79T and BsβHI-F79V showed 3.1-fold improvement of α-undecene yield. Then we focused on the alterations of identified tunnels in beneficial variants using MOLEonline server (Additional file 1: Table S3). The trends of decreased tunnel length and increased bottleneck radius were observed in the putative substrate tunnels (Tunnel 1) among variants, which is consistent with our previous assumptions. F79A variant showed the shortest substrate tunnel and the widest bottleneck radius, which were 2.8 Å decrease and 0.4 Å improvement, respectively. Interestingly, for both F79 and F173, we observed that the best decarboxylation performances were achieved by the valine substitution. The obvious feature of those two variants is the wider Tunnel 2. It is speculated that Tunnel 2 also undertakes the tunnel transfer task although this tunnel prefers polar water. Compared with Tunnel 1, Tunnel 2 has a longer length and a narrower bottleneck, which indicates that the Tunnel 2 may only serve as an auxiliary transportation for substrate. In short, the results indicated that the replacement of phenylalanine residue in positions 79 and 173 with relatively small amino acids (less bulky) had significant effect on the tunnels property and enzyme activity of P450_Bsβ_HI.

To further investigate the influence of substitution combination on the catalytic performance of P450_Bsβ_HI, we recombined the beneficial substitutions. Therefore, a series of double-site variants were constructed, which were BsβHI-F79A/F173V, BsβHI-F79S/F173V, BsβHI-F79T/F173V, and BsβHI-F79V/F173V. However, all of the double-site variants show very low α-undecene production. One possibility is that the fatty acid substrates located in the binding pocket lost the sufficient stability in the double-site variants. It was reported that the F79 and F173 residues are important for stabilization of substrate by hydrophobic interaction (Lee et al. 2003). Given that, a molecular docking simulation was carried out to examine the substrate binding affinity of P450_Bsβ_HI and its variants (Additional file 1: Table S4). Except for BsβHI-F173V, all variants showed higher binding energies and dissociation constants. For the four double-site variants, this trend is even more pronounced, especially dissociation constants. Possibly, that substrate may not be structurally complementary to the P450_Bsβ_HI active site in the double-site variants, which inhibited the catalytic activity. In other words, although the lauric acid substrate is more accessible to the binding pocket in double-site variants, the lack of strong hydrophobic interaction with F79 and F173 residues resulted in flexible and unstable binding of substrate around heme. Hence, the single-site variants of BsβHI-F79A, BsbHI-F79S, BsβHI-F79T, BsβHI-F79V, and BsβHI-F173V were chosen for further test.

### Investigation of substrate preference in P450_Bsβ_HI variants

**Fig. 4:**
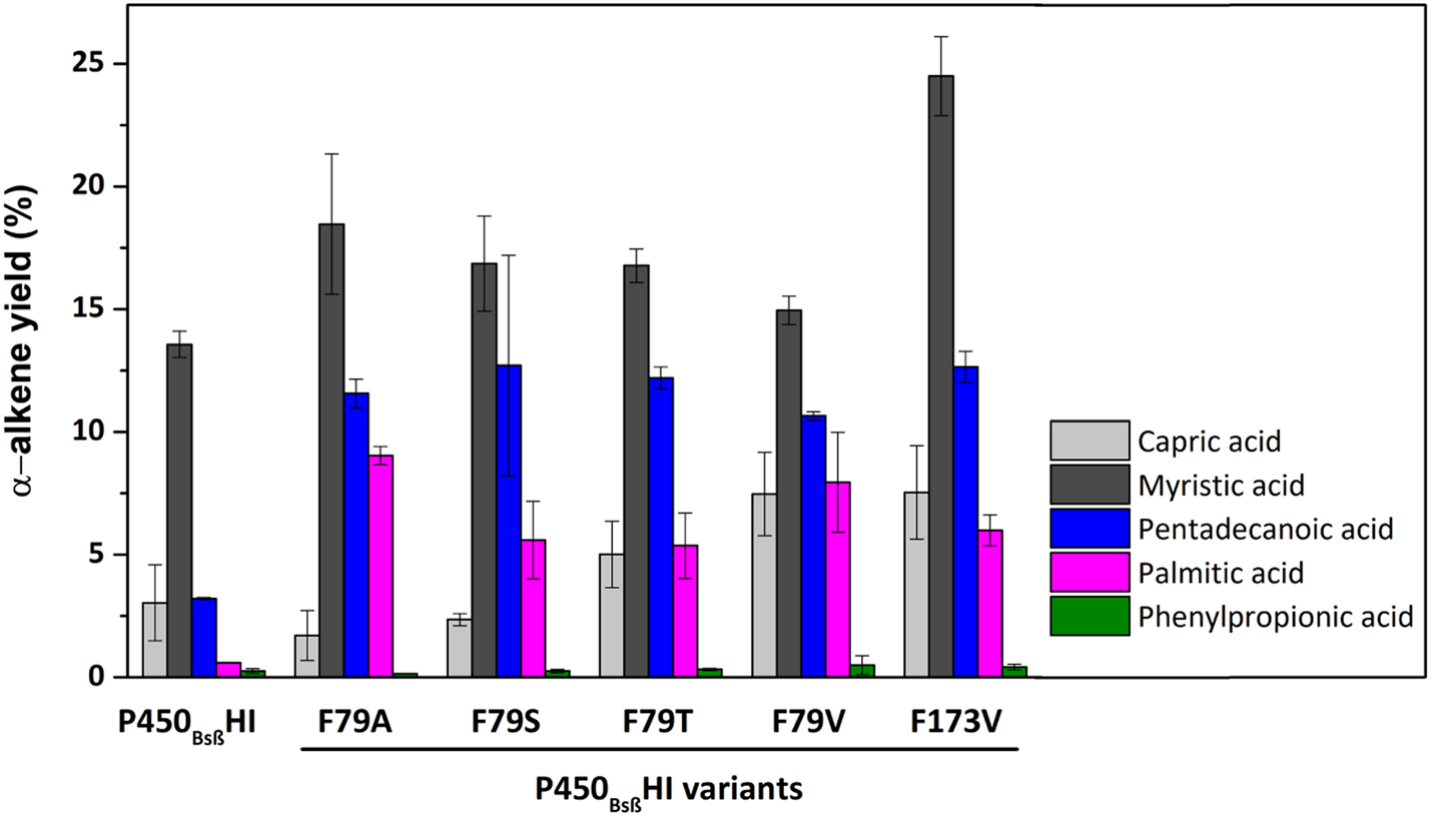
Decarboxylation performance of P450_Bsβ_HI and its variants towards various organic acid. Capric acid, myristic acid, pentadecanoic acid, palmitic acid, and phenylpropionic acid were used as substrate. All of the α-alkene products were qualitatively determined by contrasting with standard sample, and quantified by external standard method. Every bio-catalytic system contained 10 µM P450_Bsβ_HI enzyme (or its variants), 500 µM corresponding fatty acid substrate (from a 100 mM stock solution in ethanol) and 1 mM H_2_O_2_ in a final volume of 1 mL of 100 mM potassium phosphate buffer (pH = 8.0). Results shown are mean ± SD of duplicated experiments

Four additional fatty acids (capric acid, myristic acid, pentadecanoic acid, and palmitic acid) were investigated to probe the substrate profile of P450_Bsβ_HI variants and explain whether the decarboxylation activity of P450_Bsβ_HI variants is improved towards other medium- and long-chain fatty acids. In addition, given the important industrial application of styrene, phenylpropionic acid was also added for testing. As shown in Fig. 4, the decarboxylation activities of BsβHI-F79T, BsβHI-F79V, and BsβHI-F173V are higher than P450_Bsβ_HI with capric acid as the substrate, while toward long-chain fatty acids substrate, as a general trend, all candidates of P450_Bsβ_HI variants exhibit higher catalytic efficiency than P450_Bsβ_HI. Intriguingly, optimal decarboxylation efficiency toward different substrates exists in different variants. While BsβHI-F173V possesses the highest activity toward myristic acid and pentadecanoic acid, BsβHI-F79A is the best toward palmitic acid. Besides alkenes, product distribution analysis showed that the yield of hydroxyl fatty acids was also increased (Table 2). Both the variants possessed broader access tunnels than the tunnels in P450_Bsβ_HI (Additional file 1: Table S3). We reasoned that this observation may stem from the compatibility between the substrate and access tunnels. Alanine has the smallest side chain, so it provided the F79A variant the widest substrate tunnel, which is able to accommodate relatively larger fatty acid substrate, like palmitic acid. Meanwhile, hydrophobic interaction provided by nonpolar alanine or valine also reduces substrate flexibility to fatty acid substrates, leading to higher stability of enzyme–substrate complex during the reaction cycle. In addition, only trace styrene was detected when using the phenylpropionic acid as substrate, probably due to the strong preference of P450_Bsβ_HI towards fatty acid substrates (Xu et al. 2017).

**Table 2:** Regioselectivity of P450_Bsβ_HI and two best variants (BsβHI-F79A and BsβHI-F173V) towards four fatty acid substrates

Substrate	Enzyme	Conversion (%)	Product distribution (%)		
			Alkene	α-OH-FA	β-OH-FA
Lauric acid	P450_Bsβ_HI	49.5 ± 1	39	19	42
	BsβHI-F79A	65.6 ± 2	55	24	21
	BsβHI-F173V	70.8 ± 9	50	23	27
Myristic acid	P450_Bsβ_HI	28.6 ± 1	47	13	40
	BsβHI-F79A	69.2 ± 6	27	26	47
	BsβHI-F173V	71.9 ± 4	34	9	57
Pentadecanoic acid	P450_Bsβ_HI	5.4 ± 1	60	16	24
	BsβHI-F79A	44.7 ± 2	26	40	34
	BsβHI-F173V	25.3 ± 1	50	16	34
Palmitic acid	P450_Bsβ_HI	1.9 ± 1	31	6	63
	BsβHI-F79A	65.9 ± 3	14	16	71
	BsβHI-F173V	26.3 ± 3	23	4	73

## Conclusions

Overall, an access tunnel engineering was carried out to understand the substrate preference as well as for improving the decarboxylation activity and of P450_Bsβ_HI. The structure indicates that two residues (F79 and F173) locate in the bottleneck of tunnels. A series of variants of P450_Bsβ_HI were generated and investigated. Significantly improved decarboxylation activity was observed in BsβHI-F79A and BsβHI-F173V variants towards long-chain fatty acids. The results reveal that the appropriate reduction of the amino acid size at the gate of tunnels improves the enzymatic activity towards larger substrates, like long-chain fatty acids. Furthermore, our study shows that identifying and engineering key residues lining the access tunnels may be a valuable and efficient strategy for improving the performance of enzymes with buried active sites.

### Supplementary Information

**Additional file 1.** Additional Tables and Figures.

## Data Availability

All data generated or analyzed during this study are included in this article and the supplementary information file.
