# Supplementary material for: Tunnel engineering for modulating the substrate preference in cytochrome P450BsβHI
Source: Bioresour Bioprocess. 2021 Apr 3;8(1):26. doi: 10.1186/s40643-021-00379-1 (PMC10992877; doi:10.1186/s40643-021-00379-1)
Supplement: Supplementary file 1 — Additional file 1. Additional Tables and Figures. [file 40643_2021_379_MOESM1_ESM.docx]

Tunnel Engineering for Modulating the Substrate Preference in Cytochrome P450_Bsβ_HI

Shuaiqi Meng^a, b, #^, Ruipeng An^a, #^, Zhongyu Li^a^, Ulrich Schwaneberg^b, c^, Yu Ji^b^, Mehdi D. Davari^b^, Fang, Wang^a^, Meng Wang^a^, Meng Qin^a^, Kaili Nie^a^, Luo Liu^a,^ *

^1^ Beijing Bioprocess Key Laboratory, Beijing University of Chemical Technology, Beijing, 100029, PR China

^2^ Lehrstuhl für Biotechnologie, RWTH Aachen University, Worringerweg 3, 52074 Aachen, Germany

^3^ DWI-Leibniz Institut für Interaktive Materialien, Forckenbeckstraße 50, 52056 Aachen, Germany

^#^ same contribution

Tel: 0086 10 64421335

Fax: 0086 10 64416428

* To whom correspondence should be addressed. E-mail: liuluo@mail.buct.edu.cn (Luo Liu). Beijing Bioprocess Key Laboratory, Beijing University of Chemical Technology, Beijing, 100029, PR China.

**Table S1** The residues lining identified tunnels in P450_Bsβ_HI.

| Tunnel number | Residues lining in the identified tunnels |
| --- | --- |
| Tunnel 1 | H85, F79, G86, R66, L70, Q75, L293, A69, A83, G294, N82, Q352, C363, K72, Q67, N68, G290, P243, R242, G80, I170, P291, F289, N239, L78, V81, A295, A246 |
| Tunnel 2 | F173, V74, L78, L293, F292, R73, L70, H180, L42, F79, L17, G174, S77, K76, L18, C363, P243, L315, H85, G294, R242, R184, A175, P71, V176, P291, I170, L15, S14, G290, F289, N239, W181, A246 |

**Table S2** The sequence of primers for P450_Bsβ_HI mutagenesis. Mutated codon is formatted in bold red text.

| **Name** | **5’→3’ sequence** |
| --- | --- |
| BsβHI-F79G-F | TCTCTG**GGT**GGTGTTAACGCGATTCATGGTATGGATGGTTC |
| BsβHI-F79G-R | CGCGTTAACACC**ACC**CAGAGATTTCTGAACGCGTTTCGGC |
| BsβHI-F79V-F | TCTCTG**GTT**GGTGTTAACGCGATTCATGGTATGGATGGTTC |
| BsβHI-F79V-R | CGCGTTAACACC**AAC**CAGAGATTTCTGAACGCGTTTCGGC |
| BsβHI-F79A-F | TCTCTG**GCA**GGTGTTAACGCGATTCATGGTATGGATGGTTC |
| BsβHI-F79A-R | CGCGTTAACACC**TGC**CAGAGATTTCTGAACGCGTTTCGGC |
| BsβHI-F79S-F | TCTCTG**TCA**GGTGTTAACGCGATTCATGGTATGGATGGTTC |
| BsβHI-F79S-R | CGCGTTAACACC**TGA**CAGAGATTTCTGAACGCGTTTCGGC |
| BsβHI-F79I-F | TCTCTG**ATT**GGTGTTAACGCGATTCATGGTATGGATGGTTC |
| BsβHI-F79I-R | CGCGTTAACACC**AAT**CAGAGATTTCTGAACGCGTTTCGGC |
| BsβHI-F79T-F | TCTCTG**ACC**GGTGTTAACGCGATTCATGGTATGGATGGTTC |
| BsβHI-F79T-R | CGCGTTAACACC**GGT**CAGAGATTTCTGAACGCGTTTCGGC |
| BsβHI-F79C-F | TCTCTG**TGT**GGTGTTAACGCGATTCATGGTATGGATGGTTC |
| BsβHI-F79C-R | CGCGTTAACACC**ACA**CAGAGATTTCTGAACGCGTTTCGGC |
| BsβHI-F79L-F | TCTCTG**TTA**GGTGTTAACGCGATTCATGGTATGGATGGTTC |
| BsβHI-F79L-R | CGCGTTAACACC**TAA**CAGAGATTTCTGAACGCGTTTCGGC |
| BsβHI-F79P-F | TCTCTG**CCG**GGTGTTAACGCGATTCATGGTATGGATGGTTC |
| BsβHI-F79P-R | CGCGTTAACACC**CGG**CAGAGATTTCTGAACGCGTTTCGGC |
| BsβHI-F173G-F | GATTGATGCA**GGT**GGTGCGGTGGGTCCGCGCCATTGGA |
| BsβHI-F173G-R | ACCGCACC**ACC**TGCATCAATCATATCAATGAAATCGTCAGCA |
| BsβHI-F173V-F | GATTGATGCA**GTT**GGTGCGGTGGGTCCGCGCCATTGGA |
| BsβHI-F173V-R | ACCGCACC**AAC**TGCATCAATCATATCAATGAAATCGTCAGCA |
| BsβHI-F173A-F | GATTGATGCA**GCA**GGTGCGGTGGGTCCGCGCCATTGGA |
| BsβHI-F173A-R | ACCGCACC**TGC**TGCATCAATCATATCAATGAAATCGTCAGCA |
| BsβHI-F173S-F | GATTGATGCA**TCA**GGTGCGGTGGGTCCGCGCCATTGGA |
| BsβHI-F173S-R | ACCGCACC**TGA**TGCATCAATCATATCAATGAAATCGTCAGCA |
| BsβHI-F173I-F | GATTGATGCA**ATT**GGTGCGGTGGGTCCGCGCCATTGGA |
| BsβHI-F173I-R | ACCGCACC**AAT**TGCATCAATCATATCAATGAAATCGTCAGCA |
| BsβHI-F173T-F | GATTGATGCA**ACC**GGTGCGGTGGGTCCGCGCCATTGGA |
| BsβHI-F173T-R | ACCGCACC**GGT**TGCATCAATCATATCAATGAAATCGTCAGCA |
| BsβHI-F173C-F | GATTGATGCA**TGT**GGTGCGGTGGGTCCGCGCCATTGGA |
| BsβHI-F173C-R | ACCGCACC**ACA**TGCATCAATCATATCAATGAAATCGTCAGCA |
| BsβHI-F173L-F | GATTGATGCA**TTA**GGTGCGGTGGGTCCGCGCCATTGGA |
| BsβHI-F173L-R | ACCGCACC**TAA**TGCATCAATCATATCAATGAAATCGTCAGCA |
| BsβHI-F173P-F | GATTGATGCA**CCG**GGTGCGGTGGGTCCGCGCCATTGGA |
| BsβHI-F173P-R | ACCGCACC**CGG**TGCATCAATCATATCAATGAAATCGTCAGCA |

**Table S3** The properties of identified access tunnels in P450_Bsβ_HI and its variants. The access tunnel analysis was carried out with MOLEonline serve (Pravda, L, 2018). The number suffixed by the variant name is the tunnel number.

| **Variants** | **Tunnels** | **Length(Å)** | **Radius in bottleneck (Å)** | **Polarity** |
| --- | --- | --- | --- | --- |
| BsβHI | 1 | 29 | 1.4 | 4.06 |
|  | 2 | 37 | 1.2 | 8.48 |
| F79A | 1 | 26.2 | 1.8 | 4.37 |
|  | 2 | 33.8 | 1 | 12.42 |
| F79C | 1 | 27.8 | 1.6 | 6.14 |
|  | 2 | 36.8 | 1.1 | 12.42 |
| F79G | 1 | 33.2 | 1.7 | 8.37 |
|  | 2 | 30.9 | 1.3 | 14.05 |
| F79I | 1 | 31.6 | 1.4 | 3.46 |
|  | 2 | 33 | 1.3 | 11.75 |
| F79L | 1 | 29.1 | 1.6 | 4.91 |
|  | 2 | 34.6 | 1.3 | 10.94 |
| F79P | 1 | 30.6 | 1.6 | 5.45 |
|  | 2 | 35.3 | 1.3 | 10.83 |
| F79S | 1 | 27.3 | 1.6 | 4.09 |
|  | 2 | 33.8 | 1.1 | 13.04 |
| F79T | 1 | 26.7 | 1.7 | 4.62 |
|  | 2 | 33 | 1.1 | 11.3 |
| F79V | 1 | 27.4 | 1.6 | 5.03 |
|  | 2 | 34.5 | 1.3 | 11.57 |
| F173A | 1 | 27.6 | 1.6 | 6.26 |
|  | 2 | 39.1 | 0.9 | 12.26 |
| F173C | 1 | 26.6 | 1.7 | 4.01 |
|  | 2 | 34.4 | 1.3 | 11.63 |
| F173G | 1 | 28.7 | 1.2 | 7.13 |
|  | 2 | 26.6 | 0.9 | 16.99 |
| F173I | 1 | 26.3 | 1.5 | 8.82 |
|  | 2 | 34.1 | 1.3 | 12.79 |
| F173L | 1 | 32.4 | 1.4 | 5.35 |
|  | 2 | 26 | 0.9 | 14.54 |
| F173P | 1 | 26.7 | 1.6 | 7.01 |
|  | 2 | 36.8 | 1.3 | 10.79 |
| F173S | 1 | 30.1 | 1.2 | 5.52 |
|  | 2 | 32.6 | 0.8 | 14.44 |
| F173T | 1 | 27.6 | 1.6 | 6.79 |
|  | 2 | 41 | 1.3 | 10.26 |
| F173V | 1 | 28.8 | 1.7 | 6.3 |
|  | 2 | 34.5 | 1.3 | 12.81 |
| F79A-F173V | 1 | 25.5 | 1.7 | 4.35 |
|  | 2 | 37.3 | 1 | 9.5 |
| F79S-F173V | 1 | 25 | 1.7 | 4.98 |
|  | 2 | 37.3 | 1.1 | 9.49 |
| F79T-F173V | 1 | 30.5 | 1.5 | 6 |
|  | 2 | 32.2 | 1.3 | 13.8 |
| F79V-F173V | 1 | 28.4 | 1.6 | 6.97 |
|  | 2 | 34.2 | 1.3 | 11.07 |

**Table S4** Binding energies and dissociation constant determined by molecular docking of P450_Bsβ_HI and its variants with substrate lauric acid. Molecular docking simulations were performed in YASARA (version 19.12.14) using the built-in macro “dock_run.mrc”. 25 docking runs were performed for each variant, and the docking poses were clustered by applying a RMSD cutoff of 5 Å and using the default settings provided within the YASARA dock_run macro file.

| **Variants** | **Binding energy (kcal/mol)** | **Dissociation constant (μM)** |
| --- | --- | --- |
| BsβHI | - 6.41 ± 0.53 | 19.88 |
| F79A | - 6.04 ± 0.45 | 37.54 |
| F79C | - 5.51 ± 0.47 | 91.00 |
| F79G | - 5.94 ± 0.41 | 44.00 |
| F79I | - 6.00 ± 0.34 | 40.11 |
| F79L | - 5.92 ±0 .41 | 45.52 |
| F79P | - 6.08 ± 0.41 | 34.80 |
| F79S | - 6.09 ± 0.53 | 34.49 |
| F79T | - 6.04 ±0 .45 | 37.68 |
| F79V | - 5.95 ± 0.53 | 43.16 |
| F173A | - 5.66 ± 0.41 | 71.49 |
| F173C | - 5.64 ± 0.52 | 73.88 |
| F173G | - 5.70 ± 0.52 | 66.45 |
| F173I | - 6.02 ± 0.50 | 38.77 |
| F173L | - 6.15 ± 0.71 | 31.02 |
| F173P | - 5.46 ± 0.44 | 54.23 |
| F173S | - 5.85 ± 0.52 | 51.39 |
| F173T | - 5.77 ± 0.43 | 58.63 |
| F173V | - 6.73 ± 0.43 | 11.59 |
| F79A-F173V | -5.35 ± 0.45 | 85.46 |
| F79S-F173V | - 5.36 ± 0.52 | 83.31 |
| F79T-F173V | - 5.46 ± 0.42 | 70.92 |
| F79V-F173V | - 5.19 ± 0.47 | 111.98 |


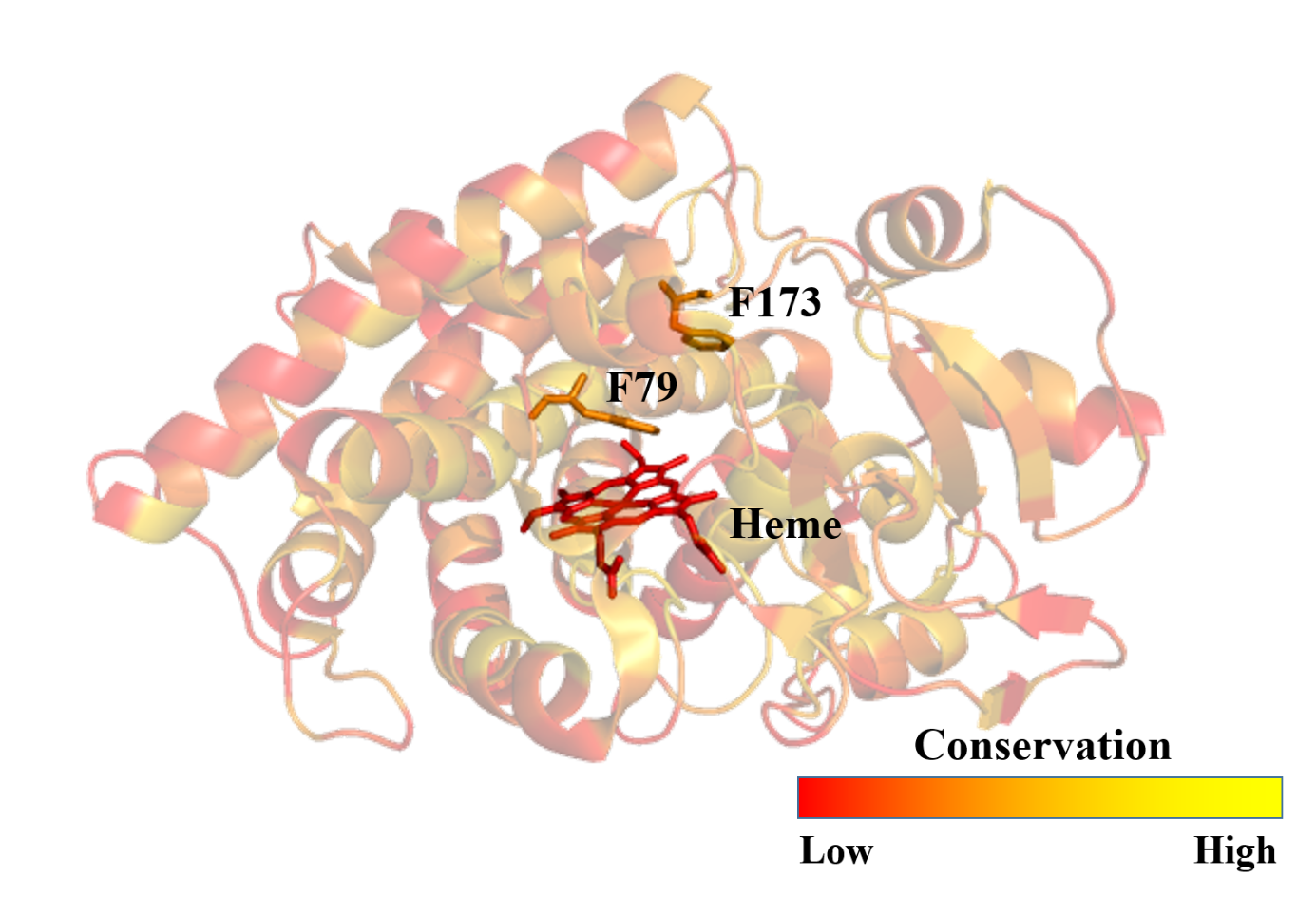


**Figure S1**: Conservation analysis of P450_Bsβ_HI. The residues of phenylalanine 79 and phenylalanine 179 were marked by green circle. The conservation analysis was performed using Consurf online serve (Ashkenazy et al., 2016). Multiple sequence alignment was built using MAFFT; The homologues were collected from UNIREF90; The homolog search algorithm is HMMER; HMMER E-value is 0.0001. The calculation is performed on P450_Bsβ_HI of 150 sequences that represent the list of homologues to the query.


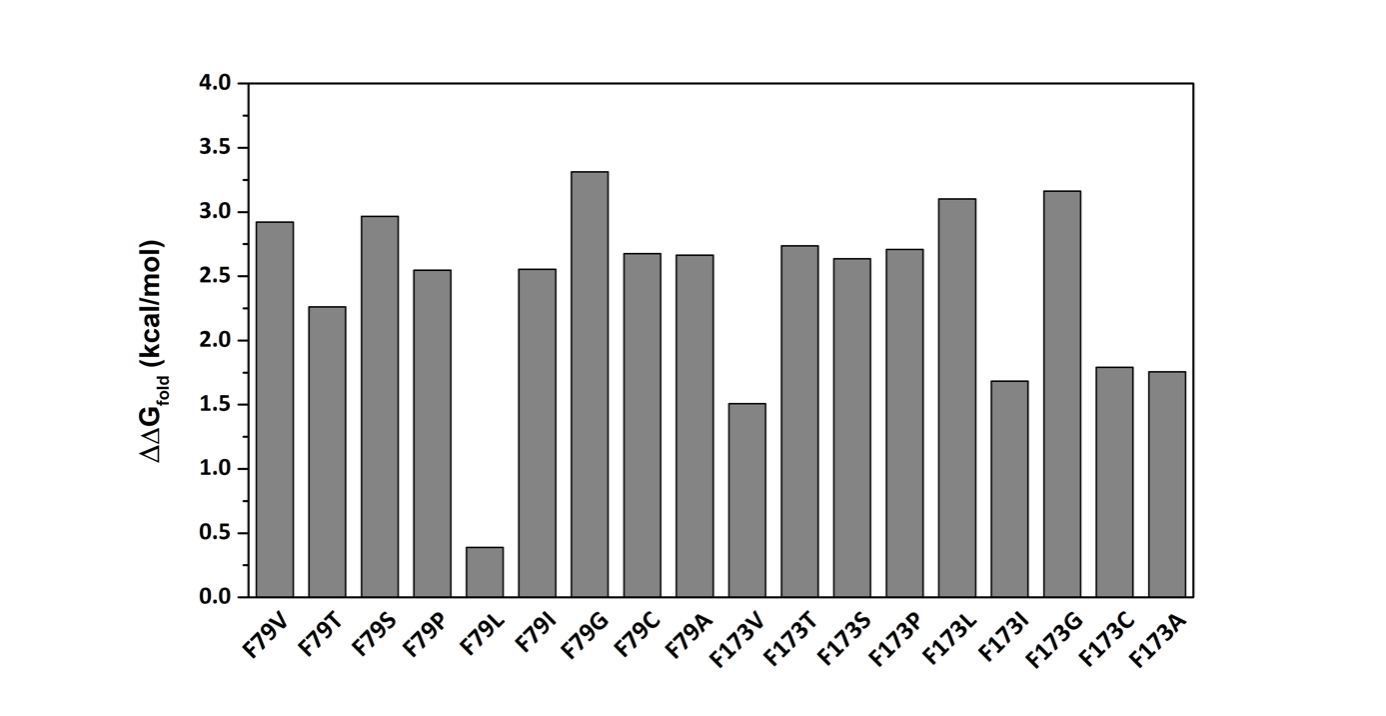


**Figure S2** The relative folding free energies (ΔΔG_fold_=ΔG_fold_,variants−ΔG_fold_,wild-type) values of 18 substitutions at two amino acid positions of the P450_Bsβ_HI.

The ΔΔG_fold_ values were calculated using FoldX employing the YASARA plugin (version 19.12.4). The initial structure of P450_Bsβ_HI was constructed by the Automated Modeling Tool of Swiss Model Web Service (http://swissmodel.expasy.org/) using the crystal structure of P450_Bsβ_ from *Bacillus subtilis* (PDB code: 1IZO with the resolution in 2.10 Å) as template. FoldX parameters was temperature 298 K, pH 7, and 0.05 M ionic strength.

Based CompassR rule developed by Cui et.al., the substitutions were divided three categories: Active (ΔΔG_fold_≤+0.36 kcal mol^−1^), Unpredictable (+0.36 kcal mol^−1^≤ΔΔG_fold_≤+7.52 kcal mol^−1^), and Inactivated (ΔΔG_fold_≥+7.52 kcal mol^−1^) [1]. There is no unstable substitution was found among all of the variants.

**Figure S3** The Root-mean-square deviation (RMSD) of CA atoms of P450_Bsβ_HI versus the starting structure.


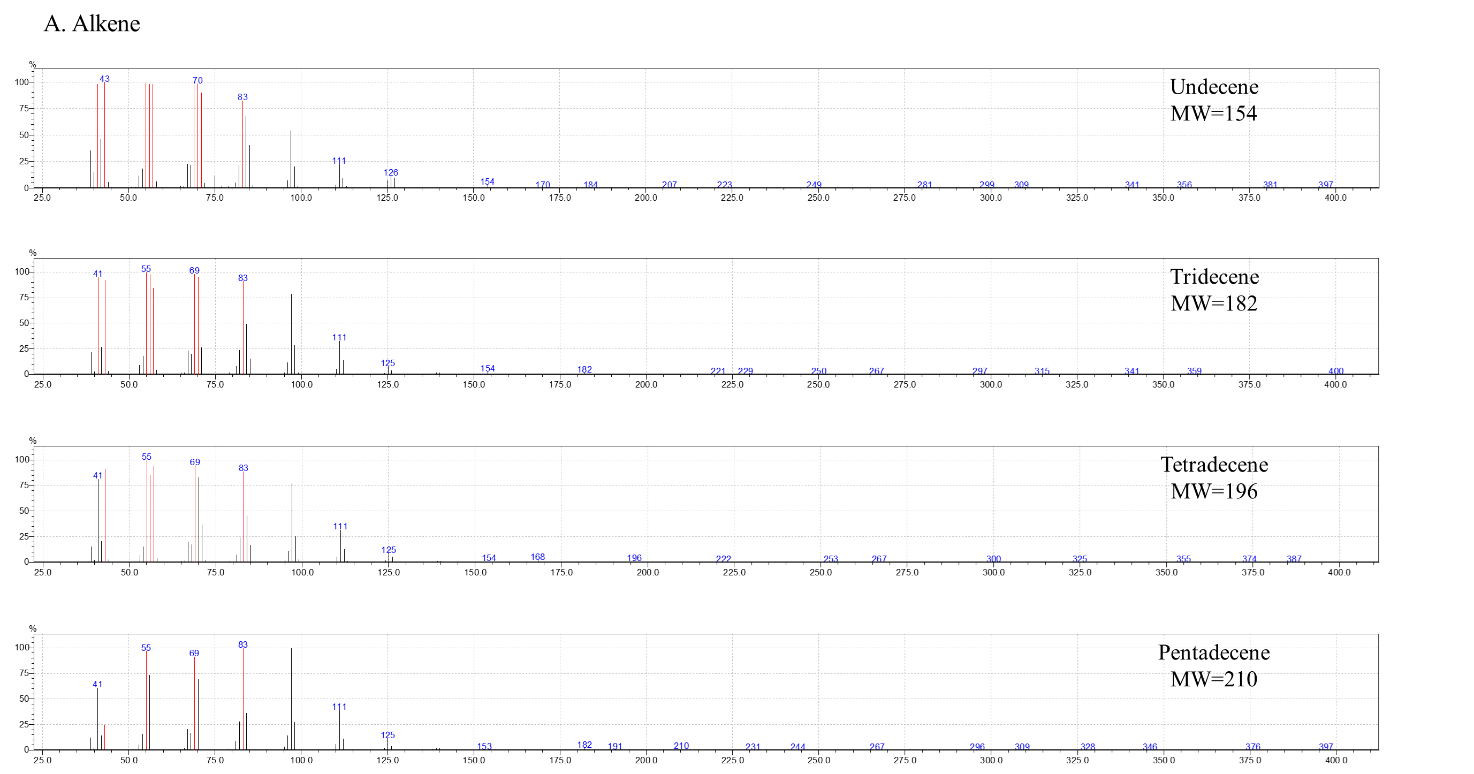


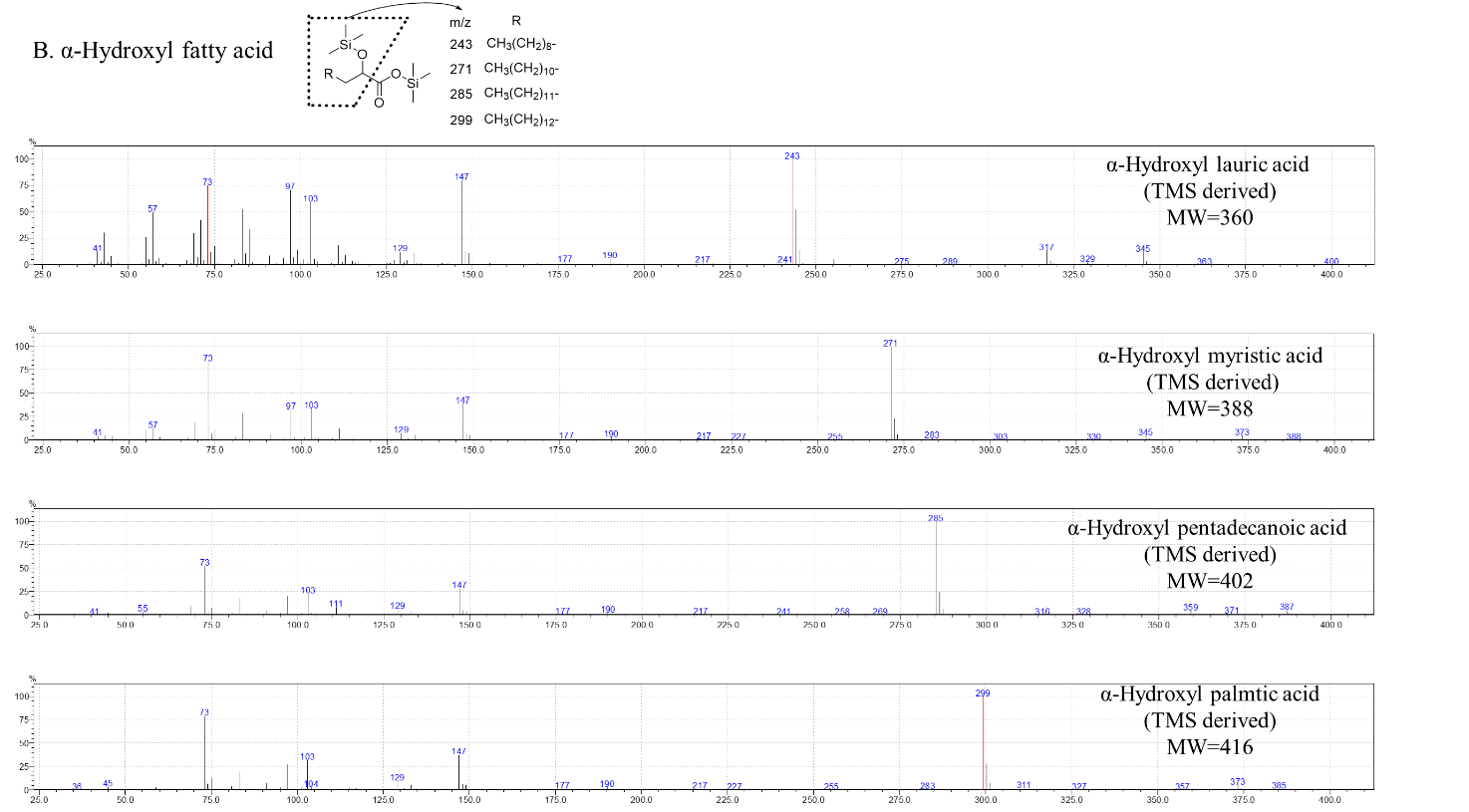


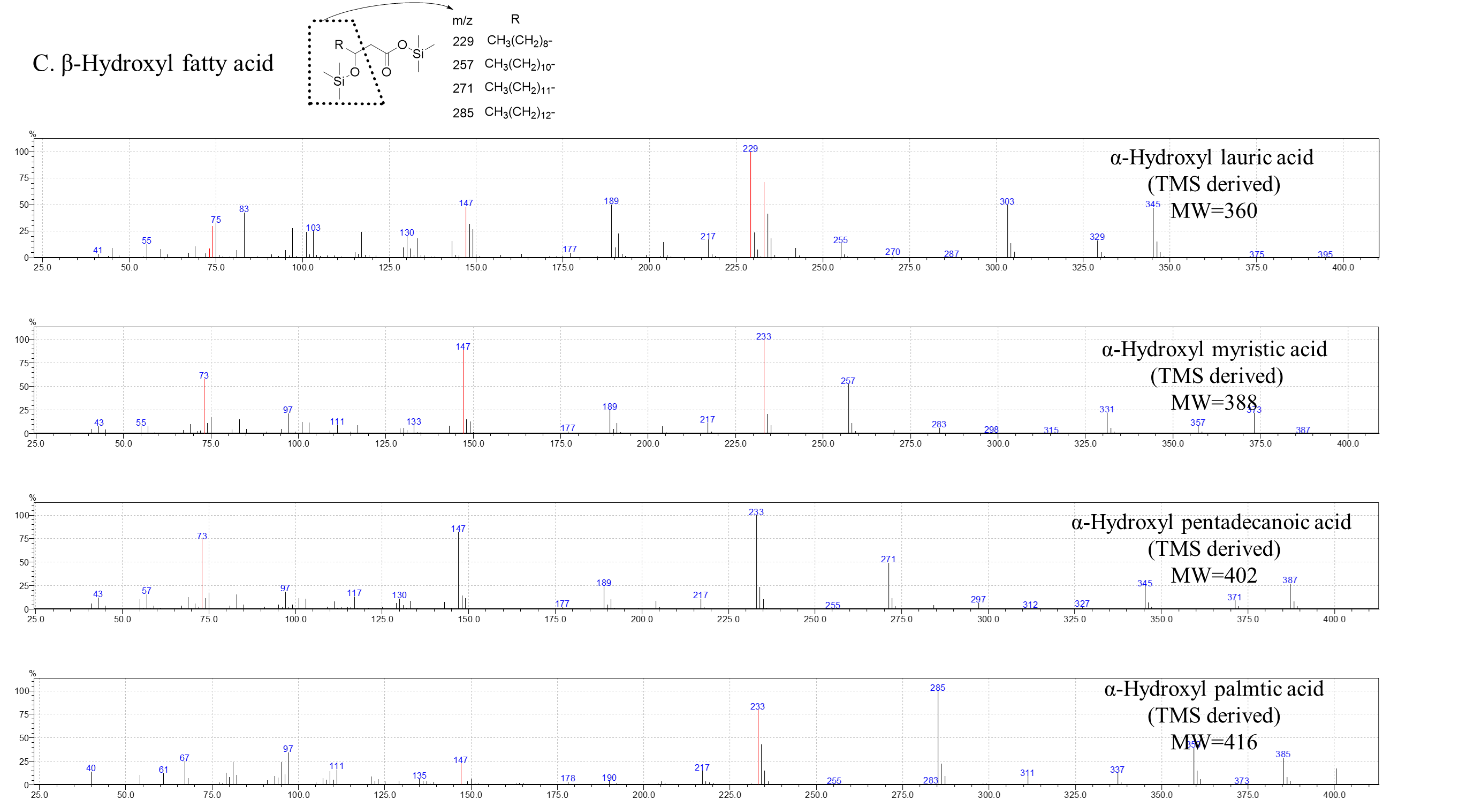


**Figure S4** Mass spectroscopic analysis of alkene and hydroxyl fatty acid products.

**
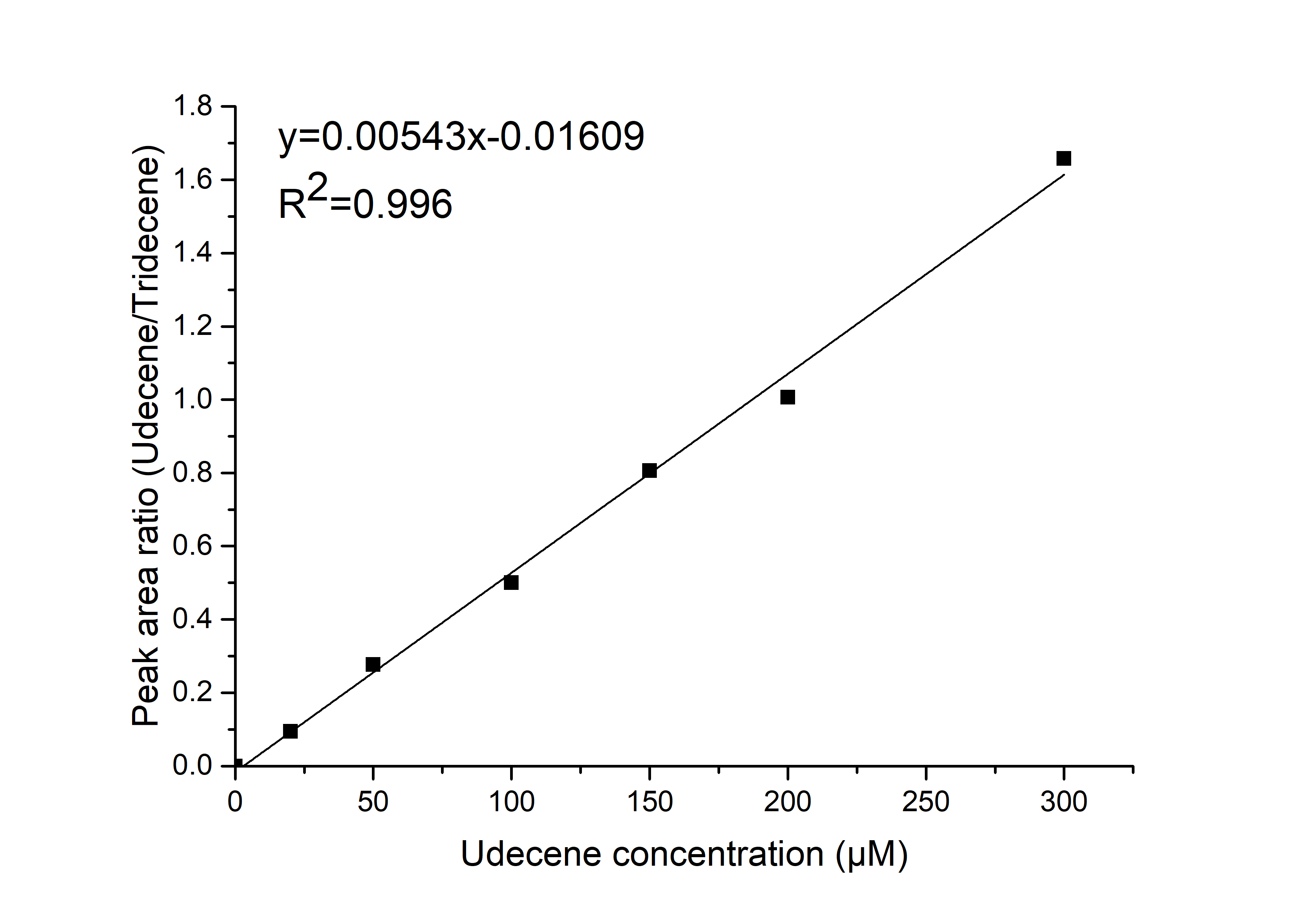
**

**
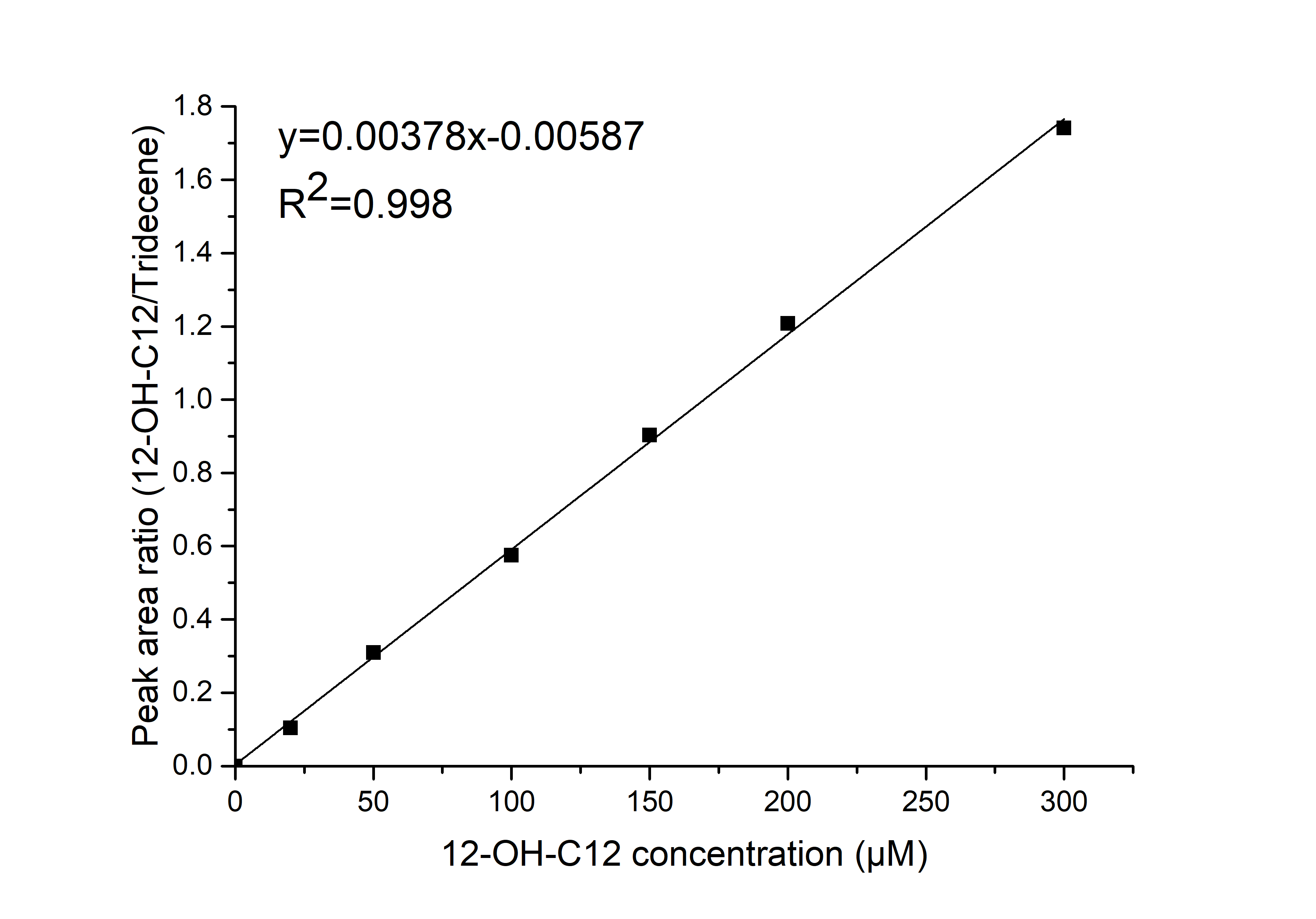
**

**Figure S5** Calibration curves for products quantification. 200 μM of tridecene was used as the internal standard. 0-300 μM of undecene (upper figure) and 12-hydroxyl lauric acid (12-OH-C12, lower figure) were used as representative compounds for quantification of alkene products and hydroxyl fatty acid products, respectively.

**Reference:**

Pravda, L., Sehnal, D., Tousek, D., Navratilova, V., Bazgier, V., Berka, K., et al. (2018). MOLEonline: a web-based tool for analyzing channels, tunnels and pores (2018 update). Nucleic Acids Res, 46(W1), W368-W373.

Ashkenazy, H., Abadi, S., Martz, E., Chay, O., Mayrose, I., Pupko, T., & Ben-Tal, N. (2016). ConSurf 2016: an improved methodology to estimate and visualize evolutionary conservation in macromolecules. Nucleic Acids Res, 44(W1), W344-W350.
